# Probabilistic Reversal Learning Deficits in Patients With Methamphetamine Use Disorder—A Longitudinal Pilot Study

**DOI:** 10.3389/fpsyt.2020.588768

**Published:** 2020-12-09

**Authors:** Maximilian Pilhatsch, Shakoor Pooseh, Alexandra Junke, Milky Kohno, Johannes Petzold, Cathrin Sauer, Michael N. Smolka

**Affiliations:** ^1^Department of Psychiatry and Psychotherapy, Technische Universität Dresden, Dresden, Germany; ^2^Department of Psychiatry and Psychotherapy, Elblandklinikum Radebeul, Radebeul, Germany; ^3^Freiburg Center for Data Analysis and Modeling, Albert-Ludwigs-Universität Freiburg, Freiburg, Germany; ^4^Department of Psychiatry, Oregon Health and Science University, Portland, OR, United States; ^5^Department of Behavioral Neuroscience, Oregon Health and Science University, Portland, OR, United States; ^6^Research and Development Service, Veterans Affairs Portland Health Care System, Portland, OR, United States; ^7^Methamphetamine Abuse Research Center, Oregon Health and Science University and Veterans Affairs Portland Health Care System, Portland, OR, United States

**Keywords:** methamphetamine, crystal meth, probabilistic, reversal learning, longitudinal, psychotherapy

## Abstract

Methamphetamine use disorder (MUD) is increasing worldwide and commonly associated with learning deficits. Little is known the about underlying trajectories, i.e., how the affected higher-order cognitive functions develop over time and with respect to abstinence and relapse. A probabilistic reversal learning (PRL) paradigm was implemented to uncover the microstructure of impulsive choice and maladaptive learning strategies in 23 patients with MUD in comparison with 24 controls. Baseline data revealed fewer optimal choices and a pattern of altered learning behavior from negative and positive feedback in patients suggesting impairments in flexibly-adapting behavior to changes of reward contingencies. Integrating longitudinal data from a follow-up assessment after 3 months of specific treatment revealed a group-by-time interaction indicating a normalization of these cognitive impairments in patients with MUD. In summary, our study demonstrates behavioral correlates of maladaptive decision-making processes in patients with MUD, which may recover after 3 months of MUD-specific therapy paving the way for further learning-based interventions. Limited by a small sample size, the results of this pilot study warrant replication in larger populations.

## Introduction

Methamphetamine use disorder (MUD) has been a growing worldwide problem, and in the last decade, the incidence in Western Europe has increased rapidly (1). Behavioral approaches have been the mainstay of treatment, and although some behavioral interventions have increased retention rates (2), little is known about predictors that influence treatment responses and the trajectories of cognitive functioning in MUD.

Dysfunctional learning processes in the pathogenesis of addictive disorders have become apparent (3), which when paired with various neurobiological sequelae associated with MA may undermine treatment efficacy. There is considerable evidence for MA-related neurobiological deficits (1, 4), including persistent gliosis and apoptosis in dopaminergic and serotonergic neurons (5) and abnormalities in morphology and function of fronto-striatal and limbic regions (6–9). Functionally, these changes have been linked to a broad range of cognitive impairments (10, 11), including multiple domains such as attention control, working memory and executive functions especially decision making (12–17).

Studies consistently show that maladaptive and impulsive decision making is common in MUD, where patients favor smaller immediate rewards over larger later rewards (12, 18–22). Although impulsive choice is influenced by the dynamic interaction of biases in delay and reward magnitude (23), it is unclear whether temporal discounting in MUD reflects deficits in processing and integrating reward contingencies and consequences. Adaptive decision making requires cognitive flexibility to maximize outcomes, whether to obtain reward or avoid punishment, and as learned drug-taking habits prevail despite devastating psychosocial consequences (24), it is critically important to identify the extent to which impulsive choice is related to deficits in processing and integrating outcomes/consequences.

Probabilistic reversal learning (PRL) paradigms enable the investigation on behavioral adaptation to changes in reward contingencies under uncertainty in the task environment (25). Thus, PRL can capture underlying deficits in behavioral flexibility (26), which requires updating choices when confronted with changes in the environment and neglecting rare events when environmental factors are stable (24). In PRL paradigms, participants must decide between two choices. Through trial-and-error, participants learn that one of the two choices is predominantly rewarded, whereas the other one is predominantly punished. The task is made more difficult by changing contingencies from time to time. Previously predominantly rewarded decisions are then more likely to lead to a punishment and vice versa. Optimal choice behavior is characterized by two strategies. First, participants should maintain the choice pattern that is predominantly associated with a reward despite the rare event of a punishment. These negative rare events must therefore be ignored. Second, participants must recognize a reversal or change in contingencies, where the former (correct) choice is predominantly punished. In this case, the strategy should be changed.

Previous studies have shown perseverative deficits in patients with a stimulant use disorder (27, 28), where difficulties in adapting behavior when contingencies change were exhibited. Another study showed that patients suffering from cocaine/crack or amphetamine dependence exhibited reduced reward-driven learning (24). In that study, stimulus-bound perseveration, a measure that shows the extent to which participants stick to their choice-making behavior regardless of the outcome, was greater in patients with substance use disorder (SUD) compared to controls. Although it is clear that SUD is associated with deficits in learning contingencies, a few studies have used PRL paradigms that can mirror these learning impairments in MUD. In addition, studies have shown that changes in cognitive function such as sustained attention can predict treatment outcomes (29, 30) or can improve with treatment (12); however, it is unclear whether addiction-specific treatment can improve longitudinal changes in cognitive flexibility and improve deficits in learning outcome contingencies in MUD.

This study, therefore, used a PRL task to investigate differences in learning mechanism associated with outcome contingencies in a sample of patients with MUD vs. healthy controls. Within the MUD group, changes in learning performance was tested before and after a 3-month addiction treatment program that included a combination of motivational interviewing, cognitive behavioral therapy, and psychoeducation. We hypothesized that patients would show an impaired ability to update their behavior whenever circumstances and contingencies changed compared to controls before treatment and that these aberrations would normalize post treatment.

## Methods

### Participants

In- and outpatients were recruited at the University Hospital Dresden. Inclusion criteria for patients with MUD were 18–65 years of age; meeting the diagnostic criteria for MA abuse or dependence according to the International Classification of Diseases (ICD-10); abstinence from illicit drug use for at least 2 days, proven with negative urine screenings for MA, amphetamines, MDMA, opioids, and THC. Only patients, for whom MA was clearly the main problem substance, were included. Exclusion criteria were any medical conditions, (e.g., schizophrenia, severe depressive symptoms, limited physical mobility) that interfere with the capability to attend group therapy, i.e., the experienced scientific staff assessed that the participants could at best be mildly affected by comorbid psychiatric symptoms.

For the control group, non-substance-abusing subjects (HCs) matched for age, sex, and education were recruited via advertisements placed on local community-based websites, which offered employment and volunteer opportunities. Participants were required to have no lifetime experience with any kind of stimulants (MA, amphetamines, MDMA, methylphenidate, cocaine, etc.) and have never been diagnosed with any psychiatric disorder including SUD. The final sample consisted of 23 MA-dependent patients and 24 HCs (Table 1). All participants provided written informed consent and received a compensation between 10 and 20€. The study was approved by the local ethics committee of the Technische Universität Dresden and carried out in accordance with the Declaration of Helsinki.

**Table 1:** Sociodemographic and clinical data of participants.

	**Patients**	**Controls**	**Statistics**
Sample size	23	24	
**Demographics**			
Sex			X^2^ = 0.28, df = 1, *p* = 0.60 (chi-square test)
Women	10 (43.5)	9 (36.0)	
Men	13 (56.5)	16 (64.0)	
Age (years)	30.4 ± 6.9	29.0 ± 5.5	*U* = 327.5, *p* = 0.41
Presence of own child	13 (56.5)	13 (52.0)	X^2^ = 0.01, df = 1, *p* = 0.75 (chi-square test)
Lower secondary school leaving certificate or less	15 (65.2)	18 (72.0)	X^2^ = 0.26, df = 1, *p* = 0.61 (chi-square test)
**Clinical data**			
MA dependence (years)	8.3 ± 5.4	n.a.	
Abstinence (days)	4.9 ± 4.0	n.a.	
Any psychiatric comorbidity	18 (78.0)	n.a.	

*Participant characteristics at baseline (T1): All tests are based on the whole sample (N = 48) and complete data on all variables. Data are number (%) or mean ± SD. Two-sided significance was assumed at p < 0.05*.

### Study Design

All study patients received treatment as usual, i.e., they completed our manual-supported methamphetamine-specific standard program, which was established at our clinic and has since been positively evaluated in terms of its effectiveness (2) and feasibility (31). This program is for patients who endorse MA as their main problem substance and who are all sufficiently motivated to change their drug consumption, i.e., to significantly reduce their use or to remain abstinent. The manual (31, 32) consists of 15 modules and includes a combination of strategies (such as motivational interviewing, cognitive behavioral therapy, and psychoeducation) and accounts for behavioral and demographic aspects specific to MA (e.g., younger users relative to other substance users, high rates of polysubstance use, frequent use in social and party settings, and the motivation to use as a performance enhancement). The effectiveness of this manual in treating MA problems has been sufficiently well-established and can be easily implemented in everyday clinical practice. It takes into account special aspects of MUD compared with other SUDs, e.g., the high proportion of young patients and polyvalent substance abuse as well as the widespread use of methamphetamine as recreational drug and for alleged performance enhancement.

Research staff, independent of providing treatment, conducted the recruitment as well as baseline (T1) and follow-up (T2) assessments after about 3 months. The assessments included a PRL task and the collection of clinical and sociodemographic data using standardized questionnaires. At T1, MA usage patterns were assessed, including age of first MA use, total duration of MA use, and days of abstinence. Psychiatric comorbidities were recorded according to ICD-10 criteria.

As described previously (12), inpatients provided weekly urine samples, and additional drug screening was performed in cases of clinical suspicion or after prolonged absences (during inpatient stay). Patients were randomly assigned to drug screening with a probability of 1/6 on working days. The cutoff for a positive result for amphetamines and MA was set at 300 ng/ml. We defined relapse as any positive screening result.

According to Petzold et al. (2), treatment was classified as “successful” if the patient attended at least 8 out of 15 group therapy sessions or was enrolled in a post-acute management program. Additionally, a single MA-positive test result during the course of treatment was allowed, provided the relapse was self-critically processed. The treatment was classified as “unsuccessful” if the therapy was prematurely terminated or if more than one relapse occurred.

### The Task

In the PRL task, participants make choices between two options and receive positive or negative feedback based on their decision. One choice has a high probability of reward (e.g., 80%) and is called the correct option, and the other, the incorrect option, most likely (e.g., 80%) delivers a punishment.

**Figure 1:**
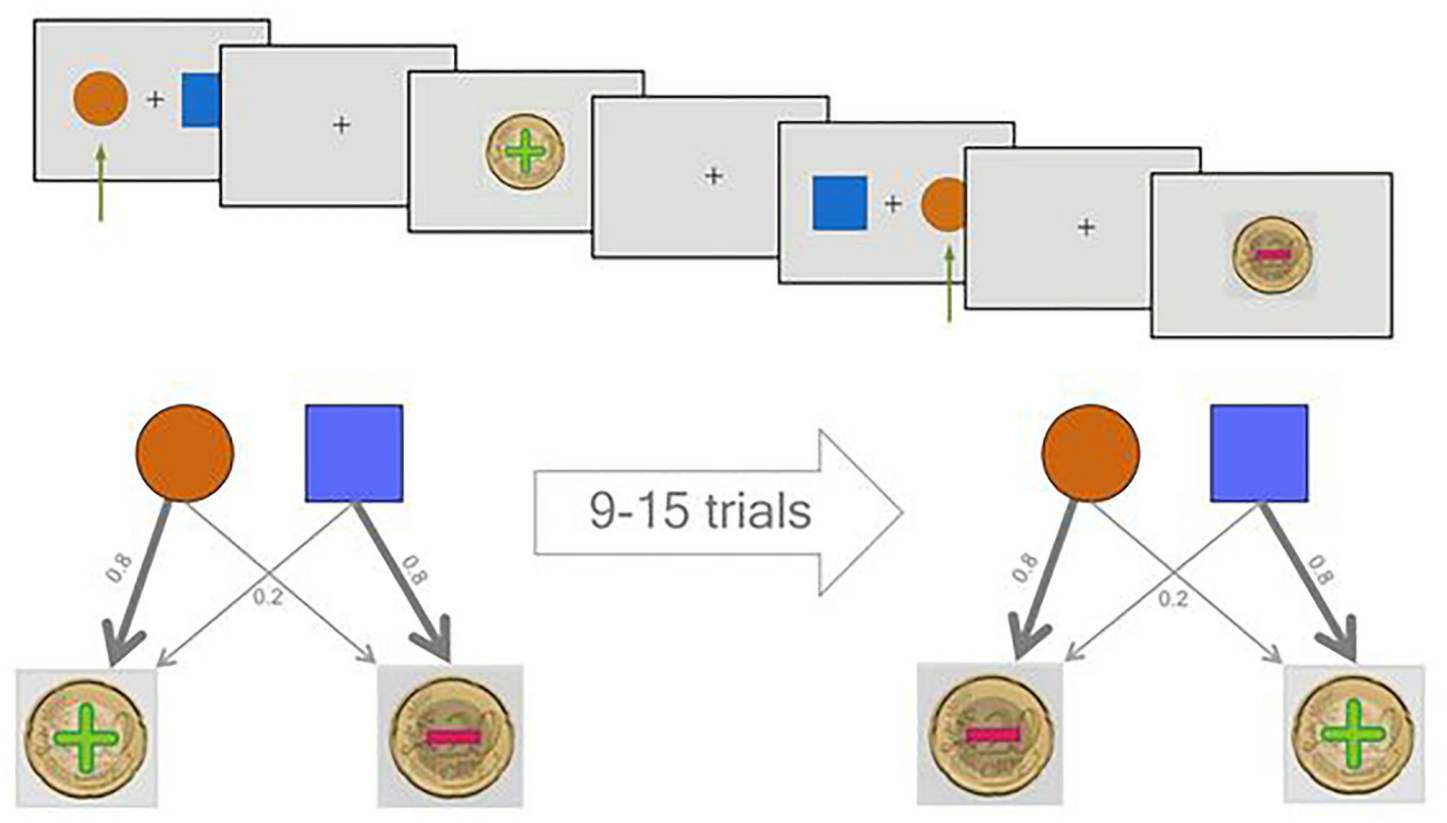
Overview of the probabilistic reversal learning (PRL) task. In the upper half of the picture, two sample trials of the task are displayed. Two sample trials are shown. The green arrow shows the selection of the participant. The first decision leads to a reward and the second to a punishment. In the lower, the probabilistic character of the task is illustrated.

Previous studies used adaptive task designs to increase the difficulty for more capable subjects. In these studies, the more rewarding option becomes the less rewarding one after a certain number of correct choices have been made (25, 33–35). For the less rewarding option, they set very close punishment and rewarding probabilities of 60 and 40%, respectively, which leads to very different expected values for different subjects. However, these modifications are too demanding for clinical populations, which frequently have cognitive impairments. To overcome these issues and make the task equally difficult for everyone, we set the reward and punishment probabilities for the correct option to 80 and 20%, respectively, and vice versa for the incorrect one (Figure 1). Moreover, we used a task design where blocks of 9–15 trials were fixed to accommodate the correct and incorrect options. We then shuffled the blocks for different participants and randomly assigned one of the cues (square or circle) to be the correct option on the first block. The reversal/contingency change was then applied such that the correct cue was switched to become the alternative cue at the beginning of each block. This design resulted in an approximately 8% chance of a contingency change from one trial to another.

Participants performed two rounds of training prior to data acquisition. The first training round was done with deterministic rewards and punishments, and the second round was a real practice with probabilistic rewards and reversals. Following the training, 11 blocks of fixed lengths were presented comprising 132 trials and 10 contingencies in total. Participants received/lost 20 cents as rewards/ punishments, displayed as Euro coins with positive and negative signs superimposed and the cumulative reward as a feedback after each trial. The task was implemented using the Psychophysics Toolbox extensions (36–38) in MATLAB, release 2017a (The MathWorks, Inc., Natick, MA).

Although different modeling techniques like reinforcement learning and hidden Markov models have been used to further investigate the mechanisms underlying decision processes, we decided on simple behavioral measures for pilot study.

### Statistical Analyses

We evaluated the differences between groups and subgroups regarding sociodemographic characteristics, clinical data, and behavioral measures with appropriate statistical tests depending on the type and distribution of variables and their sample sizes. The Shapiro–Wilk test, histograms, and normal quantile–quantile plots were used to judge normality.

For demographic and clinical data, we used Pearson's chi-square test for categorical variables, applying Fisher's exact test when needed and the unpaired *t*-test for continuous variables, applying the Mann–Whitney *U*-test when needed. Because only a few behavioral variables were normally distributed, we used the non-parametric Mann–Whitney *U*-test for all comparisons of behavioral measures for consistency.

Statistical significance was decided using an alpha level of 0.05. The exploratory longitudinal analysis was conducted using mixed effects models, which handle unequal sample sizes very well. We calculated effect sizes for the significant differences in baseline behavioral measures between controls and patients in the first measurement session.

## Results

### Sample

Clinical and sociodemographic characteristics of patients and HCs are summarized in Table 1. There were no significant differences in sex, age, and education. Among the MA group, seven were diagnosed with a cannabis use disorder, four with alcohol use disorder, two with polytoxicomania, five with borderline personality disorder, two with attention-deficit hyperactivity disorder, and three patients suffered from a depressive episode. Only one patient completed the entire study as an outpatient. Therefore, the effect of outpatient vs. inpatient treatment could not be analyzed. The duration of hospitalization was based on health insurance regulations, which cover treatment periods of 3–4 weeks for qualified drug detoxification. However, depending on comorbidities and treatment motivation, the length of stay can vary considerably. The mean duration of hospitalization was 22.5 ± 19.3 days.

Thirteen patients (56.5%) had a “successful” treatment outcome, i.e., enrollment in a post-acute management program or attending at least 8 out of 15 group therapy sessions with a maximum of one MA-positive test result. In 10 patients, the therapy was classified as “unsuccessful” since it was prematurely terminated or two or more relapses occurred (Table 2).

**Table 2:** Comparison of subsamples with successful vs. unsuccessful outcome.

	**Successful outcome**	**Unsuccessful outcome**	**Statistics**
Sample size	13 (56.5)	10 (43.5)	
**Demographics**			
Sex			*p* = 0.09 (Fisher's exact test)
Women	8 (61.5)	2 (20.0)	
Men	5 (38.5)	8 (80.0)	
Age (years)	30.1 ± 6.8	30.9 ± 7.3	*U* = 70, *p* = 0.78
Presence of own child	7 (53.8)	6 (60.0)	*p* = 1.00 (Fisher's exact test)
Lower secondary school leaving certificate or less	10 (76.9)	5 (50.0)	*p* = 0.22 (Fisher's exact test)
**Clinical data**			
MA dependence (years)	8.8 ± 6.3	7.7 ± 4.2	*U* = 61.5, *p* = 0.85
Abstinence [days]	4.2 ± 3.4	5.9 ± 4.6	*U* = 81.50, *p* = 0.31
SUD comorbidity	6 (46.2)	7 (70.0)	*p* = 0.40 (Fisher's exact test)
Psychiatric comorbidity except SUD	5 (38.5)	5 (50.0)	*p* = 0.68 (Fisher's exact test)
Treatment with antidepressant or antipsychotic	5 (38.5)	5 (50.0)	*p* = 0.68 (Fisher's exact test)
Family history of SUD	6 (46.2)	6 (60.0)	*p* = 0.68 (Fisher's exact test)

*Patient characteristics at baseline (T1): All tests are based on the whole patient sample (N = 23) and complete data on all variables. Data are number (%) or mean ± SD. Two-sided significance was assumed at p < 0.05*.

### Behavioral Measures

We considered the number of correct choices (hits), irrespective of entailing a reward or a punishment, as a measure of performance. This measure was used to compare groups and to predict clinical outcome parameters. We also compared the number of times that participants switched from or continued selecting the previous choice after losing or winning.

**Figure 2:**
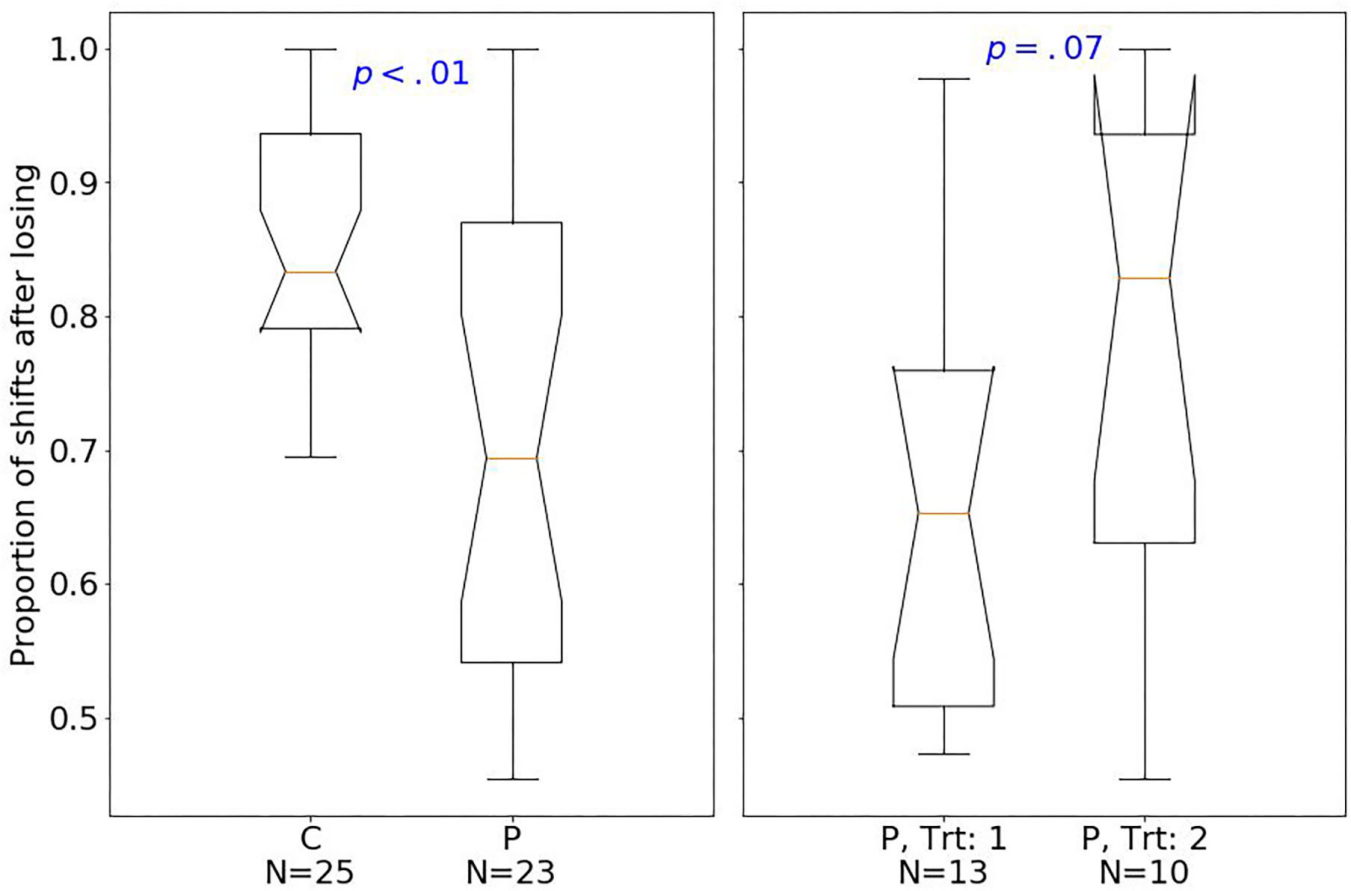
Box plots of the PRL task. The horizontal line represents the median; the boxes extend to the first and third quartile, while whiskers extend to the max/min or the corresponding quartile + 1.5 IQR. C, healthy controls; P, patients with MUD; Trt_1, successful treatment outcome; Trt_2, unsuccessful treatment outcome. Box plots of proportion of shifts after losing.

**Figure 3:**
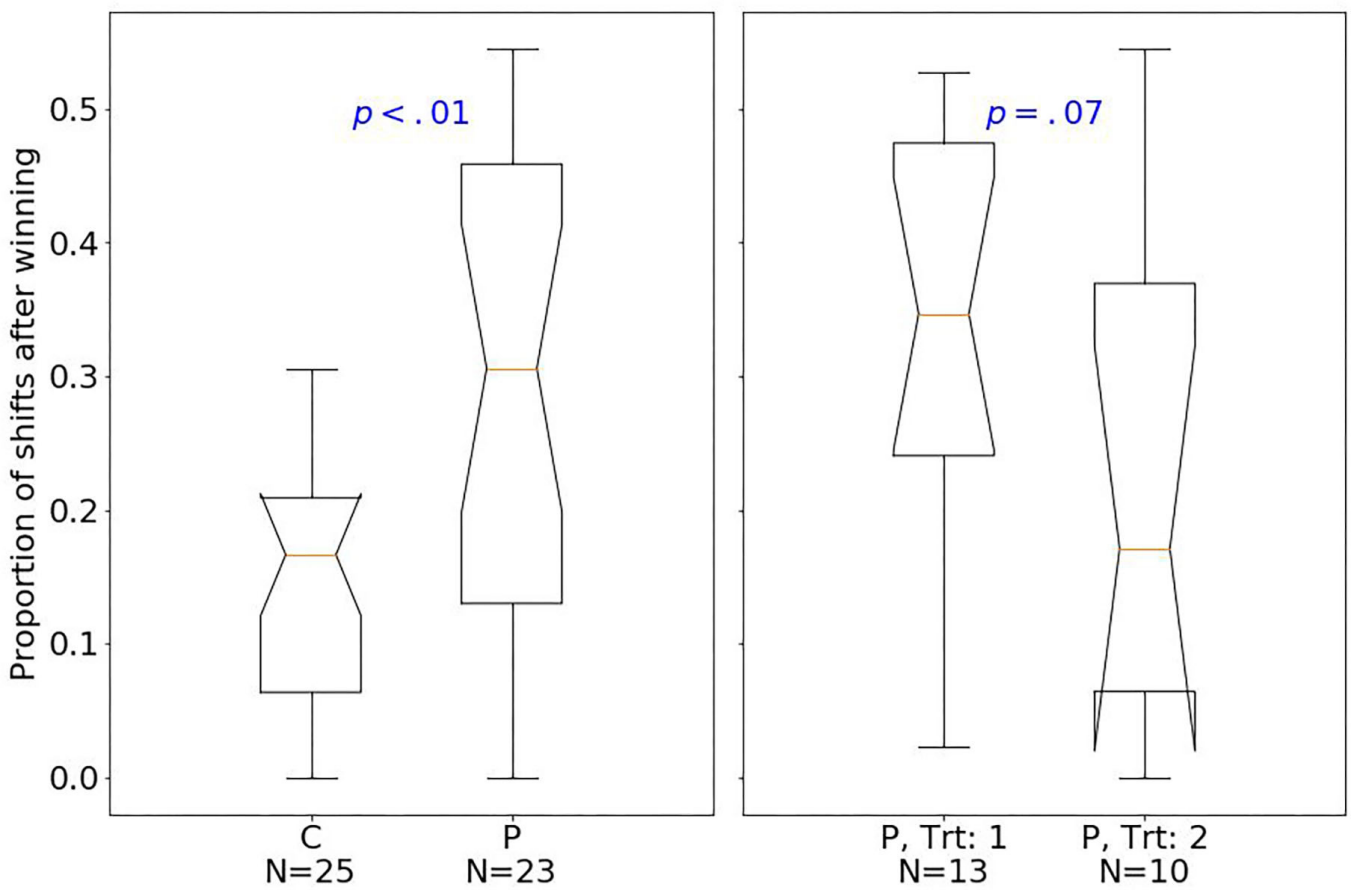
Box plots of the PRL task. The horizontal line represents the median; the boxes extend to the first and third quartile, while whiskers extend to the max/min or the corresponding quartile + 1.5 IQR. C, healthy controls; P, patients with MUD; Trt_1, successful treatment outcome; Trt_2, unsuccessful treatment outcome. Box plots of proportion of shifts after winning.

At baseline, patients had fewer hits than controls (median of 80 and 89, respectively; one-sided Mann–Whitney *U*-test, *U* = 129.5, *p* < 0.001, *r* = 0.77). The effect size quantified by Pearson's r is related to the probability that one randomly selected patient has fewer number of hits than a randomly chosen control (39). Moreover, patients had fewer shifts after losing (*p* < 0.01; Figure 2), but more shifts after winning (*p* < 0.01; Figure 3) compared with controls.

**Figure 4:**
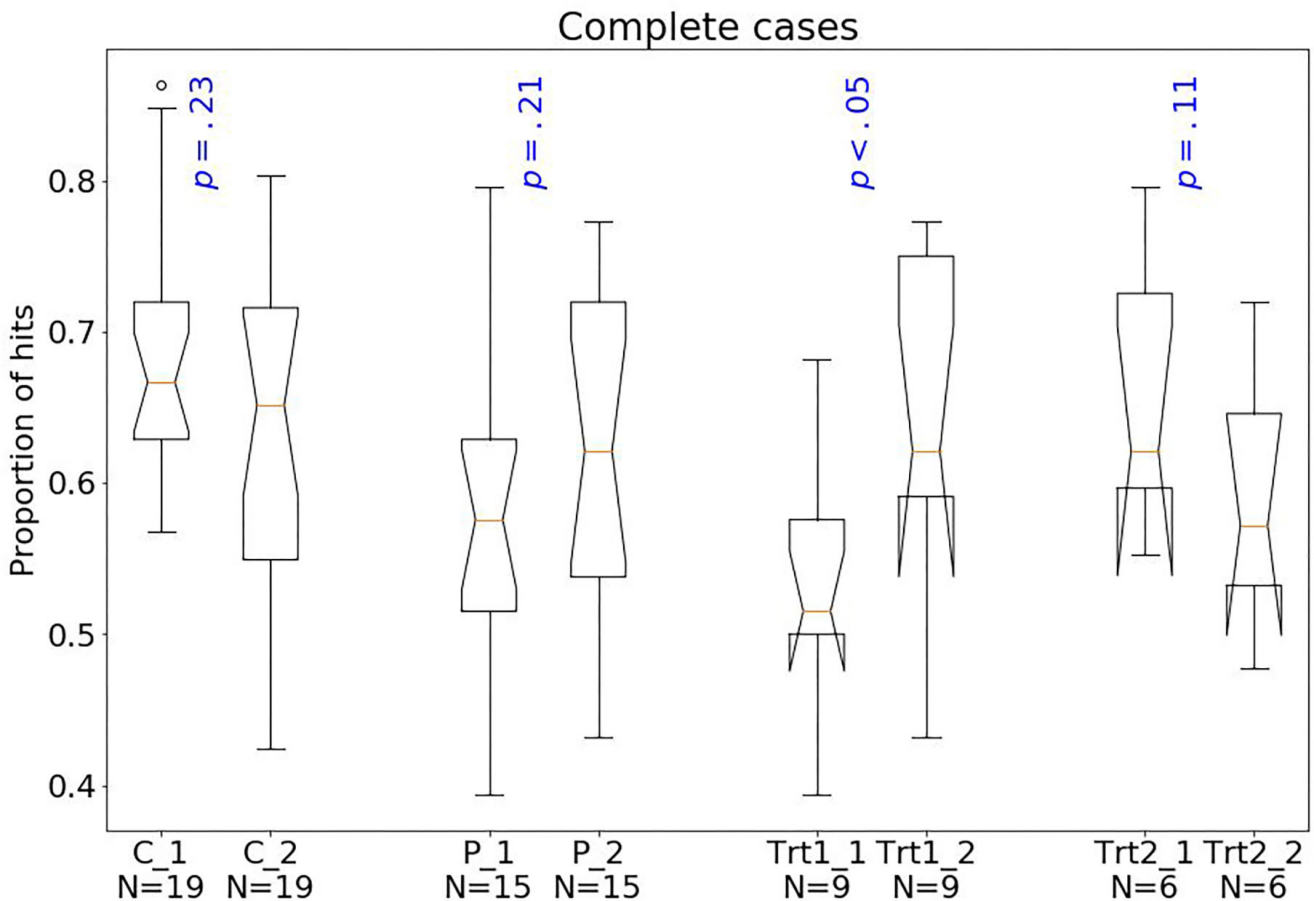
Box plots of the PRL task. The horizontal line represents the median; the boxes extend to the first and third quartile, while whiskers extend to the max/min or the corresponding quartile + 1.5 IQR. C, healthy controls; P, patients with MUD; Trt_1, successful treatment outcome; Trt_2, unsuccessful treatment outcome. Box plots of proportion of hits.

Considering treatment outcomes, patients with a successful treatment had fewer hits (*p* < 0.01) than those with an unsuccessful one (Figure 4) and also tended to have fewer shifts after losing (*p* = 0.07; Figure 2) and more shifts after winning (*p* = 0.07; Figure 3) at baseline.

### Longitudinal Analysis

**Figure 5:**
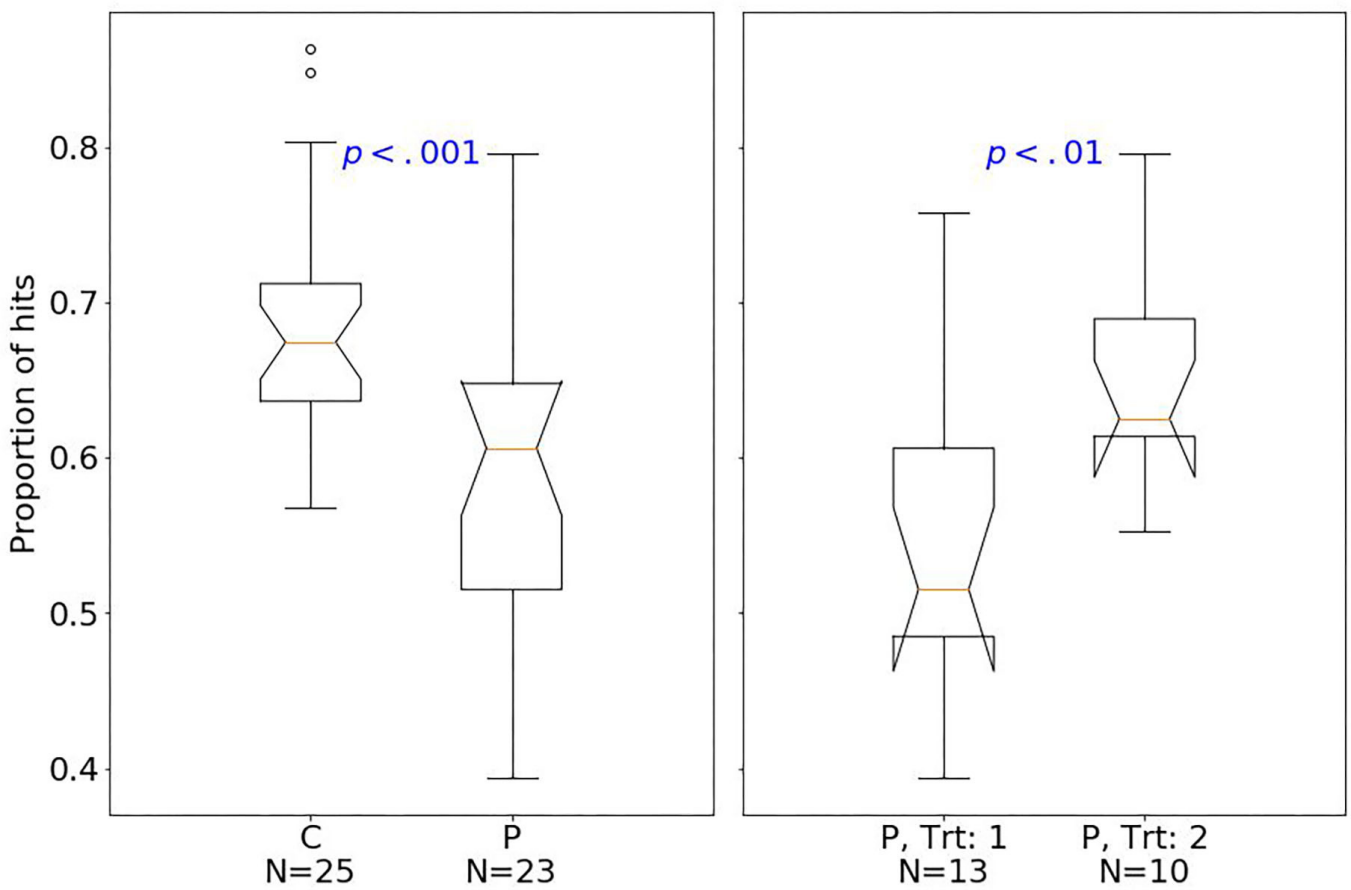
Box plots of the PRL task. The horizontal line represents the median; the boxes extend to the first and third quartile, while whiskers extend to the max/min or the corresponding quartile + 1.5 IQR. C, healthy controls; P, patients with MUD; Trt_1, successful treatment outcome; Trt_2, unsuccessful treatment outcome. Box plots of proportion of hits.

Follow-up data at T2 could be acquired for 76% of controls and 65% of patients leading to a considerable reduction in the statistical power. Nevertheless, we performed an exploratory longitudinal analysis using mixed effects models to investigate effects of time, group, and their interaction. The analysis was done with *nlme* package in R (40). As shown in Table 3, we found a significant group effect and a trend for the interaction term: The main effect of group (*p* = 0.001) indicated that patients exhibited a lower number of hits than controls. The almost significant time-by-group interaction (*p* = 0.057) indicated a possible improvement in the number of hits from T1 to T2 in patients but not in HC (Figure 5). We further conducted a multiple imputation, using the *mice* package in R (41), which suggests that the group effect is consistent, and statistically significant interaction effects might be found with a higher sample size (Table 4).

**Table 3:** Output of mixed effects model using nlme package in R.

	**Value**	**Std. error**	**DF**	***t*-value**	***p*-value**
(Intercept)	65.77	5.91	46	11.13	0.000
Time	14.28	8.50	32	1.68	0.103
Group	12.54	3.69	46	3.40	0.001
Time:group	−10.31	5.23	32	−1.97	0.057

**Table 4:** The 95% confidence intervals from multiple imputation using mice package in R.

	**2.5%**	**97.5%**
Time	−7.79	26.37
Group	5.21	19.86
Time:group	−17.71	3.35

## Discussion

In this study, a typical clinical sample of German patients with MUD demonstrated a lower number of optimal choices than healthy controls in a PRL paradigm suggestive of overall maladaptive decision making. Patients showed greater difficulties in adjusting their behavior following a rule change compared with HC. This finding is in line with earlier studies suggesting similar deficits in patients suffering from amphetamine (24, 27) and cocaine (42) dependence from the United Kingdom. Using a combination of a “Go/No-Go” task with reversal learning, a study has shown maladaptive choices among cocaine and alcohol-dependent patients when contingency change. Here, the patients tended to increase stimulus-bound perseveration (43).

To differentiate the microstructure of behavior, we analyzed specific learning patterns from positive and negative reinforcement. Compared with controls, patients exhibited stimulus-bound perseveration in low-reward probability choices, where they maintained their choices after losing. Interestingly, patients with MUD continued to choose the wrong option despite the higher probability of receiving negative consequences but shifted their response to the wrong option after winning. In contrast, controls switched to the other option after losing more often than patients (“lose-shift”) and more often stayed at the same option after winning (“win-stay behavior”). Intuitively, a reasonable strategy is to change response patterns after receiving a punishment and to maintain it after receiving a reward. To that extent, the responding pattern of patients can be considered closer to random decision making.

This learning pattern is in line with that of Ersche et al. (42), who found that individuals with cocaine dependence were impaired in both learning from negative and positive reinforcement. Kanen et al. (24) also showed diminished win-stay behavior in SUD; however, learning from negative feedback (lose-shift) was exhibited in SUD. The difference in results might be influenced by the salience of feedback. This study and the study of Ersche et al. implemented aversive negative outcomes; the loss of money or electric shock (respectively), while the feedback in Kanen et al. was an image of a sad red face, which presumably engenders less motivation. These results suggest the importance of salient and motivating punishments to drive learning and behavior. From a clinical perspective, impaired learning capacities from high-salient negative consequences highlight the ineffectiveness of punitive, interventions for SUDs.

### Longitudinal Course

An analysis of the longitudinal course showed a main effect of group and an almost significant (*p* = 0.057) group-by-time interaction. After imputing missing values for T2, the interaction became significant. While the number of hits was not significantly different between T1 and T2 in controls, the performance improved in patients over time but did not reach statistical significance; however, the significant differences in performance between HC and patients with MUD at T1 were ameliorated at T2. The improvements in the MUD group were primarily driven by patients with better treatment outcomes. The almost significant group-by-time interaction and the lack of behavioral differences between groups at T2, suggests that the ability to update behavior when circumstances change can normalize in patients with MUD after 3 months of specific therapy. Since the sample size is small and some values had to be imputed, this interpretation should be treated with caution and requires confirmation in larger studies. On the other hand, the results fit plausibly to the existing data: Bernhardt et al. (12) showed that sustained attention deficits in patients with MUD could also normalize over a 3-month therapy period. Along those lines, Volkow et al. (17) and Wang et al. (44) found an improvement in motor and verbal memory. Moreover, even in short observation periods (i.e., 3 weeks), the performance of patients with MUD in neuropsychological tests including attention (45) and executive functioning (46) showed an improvement. Limited by the lack of a control group, effects on the causal role of abstinence on performance changes can hardly be concluded (12). In another longitudinal study, however, MA-dependent participants showed a normalization of global cognitive function compared to control subjects after an average abstinence of 1 year (47). These results might question the usefulness of treatment strategies based on contingency management at an early stage. Contingency-based strategies in the treatment of MUD may be more successful once behavioral control through reward and punishment contingencies has normalized. Our study suggests that this could be the case after 3 months.

### Predictors

Next, we explored whether certain patterns in PRL performance at T1 were associated with clinical outcome parameters. For this purpose, the group of patients was divided into “successful” (*n* = 13; 56.5%) and “unsuccessful” (*n* = 10) depending on the clinical outcome. This rate of successful courses is in the upper range of previously investigated, comparable therapies with success rates between 30 and 70% (2). This could be due to small sizes but could also reflect that targeting MA-specific behavior is an effective approach in intervention strategies. Various particularities of MA dependence compared to other substances are taken into account, including the young age of the persons concerned, the relation to the party scene, and the high importance of other addictive substances, and this approach should be extended in future studies with sufficient sample sizes. A comparison between both groups did not reveal any significant differences in demographic or clinical parameters, but the response pattern differed significantly and revealed unexpected results. Participants with unsuccessful treatment outcomes showed significantly better performance in the “proportion of hits” at T1 compared to participants with successful treatment outcomes and were on par with the performance of healthy controls. This pattern was also significant when comparing proportion of shifts after winning and shifts after losing. These effects were relatively large and constant in different analyses. To our knowledge, such a result has not yet been described in the literature.

It could be speculated that baseline impairments in decision making is not a predictor in treatment success, but rather, the cognitive capacity for improvement is important to sustain abstinence. The research, however, on baseline cognitive performance as a predictor for treatment outcome is mixed. In a study of alcohol use disorder, the baseline performance on a probability discounting task did not predict treatment outcome (48), whereas baseline executive function predicted treatment retention in other studies (49–51). Another study showed that a subset of cognitive tasks can predict treatment success but not an overall composite score of cognitive performance (52). Notably, the relationship between cognition and abstinence was shown to be mediated by the improvements in coping skills learned in cognitive behavioral therapy sessions (53). As the successful treatment group in this study performed worse at baseline but showed the greatest improvement over time, the results suggest that perhaps, cognitive enhancement in PRL translates to other domains of behavioral control. This is in line with the goal of behavioral therapy to enhance learning skills that strengthen cognitive control to maintain abstinence (54, 55). The ability to flexibly adapt and learn new contingencies may be an important component of abstinence, as animal models of extinction show reductions in cue-elicited or drug-seeking responses in animals that have learned new associations with drug administration (56). Together the data suggest that the capacity for inhibitory control and learning new contingencies may reduce relapse. With a growing number of pharmaceutical agents for addiction designed to enhance cognitive performance (54), therapy that improves cognitive flexibility, in combination with medications may facilitate the requisite behavioral change needed to maintain abstinence. Another possibility is that individual variability of abstinence on brain function may drive differences in cognitive performance. Future studies examining these possibilities will greatly enhance treatment approaches for MUD and other use disorders.

Future studies examining these possibilities will greatly enhance treatment approaches for MUD and other use disorders.

### Limitations

First, the sample size was small, and the sample had various comorbid conditions including multidrug abuse. Therefore, the study had limited power for between and within-subject analyses making it difficult to determine whether the decision-making impairments and progresses are related to comorbidities. In addition to other SUDs, depression, for example, can also change PRL (57). Second, no control condition for the MA-specific intervention was included. Therefore, we cannot differentiate specific therapeutic effects from unspecific abstinence effects.

On the other hand, our study has several strengths, exemplified by the longitudinal control group and a naturalistic sample of MA patients with comorbid psychiatric disorders and drug abuse histories.

## Conclusion

Our study demonstrates behavioral correlates of maladaptive decision-making processes and an imbalanced learning from negative and positive feedback in patients with MUD. Because these perturbations may recover after 3 months of MUD-specific therapy, our findings warrant the development of further learning-based treatments.

## Data Availability Statement

The raw data supporting the conclusions of this article will be made available by the authors, without undue reservation.

## Ethics Statement

The studies involving human participants were reviewed and approved by the study was approved by the local ethics committee of the Technische Universität Dresden and carried out in accordance with the Declaration of Helsinki. The patients/participants provided their written informed consent to participate in this study.

## Author Contributions

MP and MS designed the study. SP and MS developed the PRL task. AJ, CS, and MP contributed to study management, data collection and processing. AJ, SP, JP, MK, and CS analyzed the data. SP, MP, JP, AJ, MK, and MS wrote the manuscript. MK, MP, JP, and SP processed the revision process. MK thoroughly revised the language of the manuscript. All authors listed have made a substantial, direct and intellectual contribution to the work, approved it for publication, and reviewed the final manuscript.

## Conflict of Interest

The authors declare that the research was conducted in the absence of any commercial or financial relationships that could be construed as a potential conflict of interest.
